# Retraction: Role of the PEBP4 protein in the development and metastasis of gastric cancer

**DOI:** 10.18632/oncotarget.28875

**Published:** 2026-04-17

**Authors:** Zijian Wu, Bin Liu, Xuemin Zheng, Huijing Hou, Ying Li

**Affiliations:** ^1^Tianjin Key Laboratory of Food Biotechnology, College of Biotechnology and Food Science, Tianjin University of Commerce, China; ^2^Tianjin Institute of Pharmaceutical Research, China; ^3^Tianjin Neurological Institute, Tianjin Medical University General Hospital, China; ^*^These authors have contributed equally to this work

**This article has been retracted:** Oncotarget has concluded the investigation of this paper. Following a request from the authors to retract the article due to “unrepeatable experiments,” the journal discovered that Figure 1, Panel B (GAPDH), was duplicated from Figure 2, Panel B (b-actin), of an article published in another journal the previous year [1]. Authorship is not shared between the two publications. The duplication involved rescaling and adjustments to brightness and contrast.

The authors’ institutions were notified of the retraction request but did not respond. Additionally, the authors were asked to provide explanations regarding the non-reproducible data and the image duplication, but they also failed to reply.

Consequently, the Editorial decision has been made to retract the article.

Original article: Oncotarget. 2017; 8:18177–18184. 18177-18184. https://doi.org/10.18632/oncotarget.15255
